# The Design of the Ni_3_N/Nb_4_N_5_ Heterostructure as Bifunctional Adsorption/Electrocatalytic Materials for Lithium–Sulfur Batteries

**DOI:** 10.3390/nano15131015

**Published:** 2025-07-01

**Authors:** Xialei Li, Wen Shang, Shan Zhang, Chun Xu, Jiabiao Lian, Guochun Li

**Affiliations:** 1 Key Laboratory of Fine Chemical Application Technology of Luzhou, Sichuan Vocational College of Chemical Technology, Luzhou 646300, Chinazhangshan@sccc.edu.cn (S.Z.);; 2 Key Laboratory of High-Tech Research on Power Batteries and Energy Storage of Zhenjiang, Institute for Energy Research, Jiangsu University, Zhenjiang 212013, China; jblian@ujs.edu.cn; 3 Huizhou Research Institute, Sun Yat-sen University, Huizhou 516081, China

**Keywords:** lithium–sulfur batteries, heterostructure, modified separator, adsorption, electrocatalysis

## Abstract

Lithium–sulfur (Li-S) batteries are hindered by the sluggish electrochemical kinetics and poor reversibility of lithium polysulfides (LiPSs), which limits their practical energy density and cycle life. In order to address this issue, a novel Ni_3_N/Nb_4_N_5_ heterostructure was synthesized via electrospinning and nitridation as a functional coating for polypropylene (PP) separators. Adsorption experiments were conducted in order to ascertain the heterostructure’s superior affinity for LiPSs, thereby effectively mitigating their shuttling. Studies of Li_2_S nucleation demonstrated the catalytic role of the substance in accelerating the deposition kinetics of Li_2_S. Consequently, Li-S cells that employed the Ni_3_N/Nb_4_N_5_-modified separator were found to achieve significantly enhanced electrochemical performance, with the cells delivering an initial discharge capacity of 1294.4 mAh g^−1^ at 0.2 C. The results demonstrate that, after 150 cycles, the cells retained a discharge capacity of 796.2 mAh g^−1^, corresponding to a low capacity decay rate of only 0.25% per cycle. In addition, the rate capability of the cells was found to be improved in comparison to control cells with NiNb_2_O_6_-modified or pristine separators.

## 1. Introduction

The pursuit of high-energy-density, sustainable energy storage technologies has prompted extensive research into metal–air batteries (MABs) and lithium–sulfur (Li-S) batteries as promising alternatives to conventional lithium-ion batteries (LIBs). Although theoretical analyses indicate that MABs, including lithium–air (Li-air) and sodium–air (Na-air) systems, possess the potential to exhibit exceptional energy densities, their practical deployment remains constrained by critical challenges [1,2,3,4].

In contrast, Li-S batteries offer a compelling balance of theoretical energy density (2600 Wh/kg, six times higher than lithium-ion (Li-ion) batteries), material abundance (sulfur is inexpensive and abundant in the earth’s crust), and environmental benignity [5,6]. However, several critical challenges remain unresolved. Among these, the design of sulfur-containing host materials has been demonstrated as an effective strategy to enhance cycling stability, as evidenced by extensive studies [7,8,9,10]. Numerous host materials, such as metal oxides [11,12], sulfides [13], and nitrides [14,15,16], as well as metallic species with distinct dispersion states (e.g., nanoparticles [17,18,19] and single-atom catalysts [20,21]), have exhibited catalytic effects in facilitating the rapid and efficient conversion of long-chain lithium polysulfide to Li_2_S. It is well known that the size of the electrocatalyst significantly affects its catalytic activity in non-homogeneous catalysis, there is no theory to explain and predict the behavior of electrocatalysts with different particle sizes in different reactions, and the dependence of the kinetics of sulfur redox electrocatalysis on the degree of aggregation of the electrocatalyst atoms is yet to be explored [22], which is also crucial for understanding the underlying basis of electrocatalysis in Li-S batteries.

Since Manthiram et al. introduced the concept of employing functional interlayers or coatings between the cathode and separator to suppress the polysulfide shuttle effect and enhance sulfur utilization in 2012 [23], significant efforts have been dedicated to developing advanced interlayer materials. Transition metal compounds [24,25,26,27,28,29,30,31,32,33,34] (e.g., oxides, nitrides, sulfides, and selenides) have emerged as promising candidates due to their strong polar-polar interactions with LiPSs and superior chemisorption capabilities [35], thereby effectively improving cell stability.

In some cases, materials with identical chemical compositions may exhibit significantly distinct charge transfer mechanisms or thermodynamic properties, primarily governed by particle size variations [23]. Electrodes composed of smaller particles generally demonstrate higher electrical conductivity, which is critical for minimizing ohmic losses during cycling [36]. Furthermore, reducing the particle size of electrode materials typically enhances capacity retention at elevated current rates. This phenomenon can be rationalized through Fick’s first law of diffusion and stochastic process theory [37], where the diffusion length is expressed as L∝$\sqrt{D\times t}$, with D representing the diffusion coefficient of charge carriers and t denoting the characteristic diffusion time. Decreasing the particle size effectively reduces the diffusion time required for lithium-ion intercalation into the electrode bulk, thereby improving charge–discharge kinetics.

In recent years, heterojunction engineering has emerged as a promising strategy for modulating the electronic structure of catalytic materials. This approach leverages the unique capability of heterojunctions to regulate band structures, accelerate charge transfer, and enhance catalytic conversion from LiPS (lithium polysulfides) to Li_2_S. Table 1 lists some recent heterojunction applications of the interlayers in Li-S batteries. As seen, Li et al. [38] synthesized a WS_2−x_/ZnS@C composite, where the constructed heterojunction not only facilitates charge transfer and ion diffusion but also improves reaction kinetics, thereby boosting overall electrochemical performance. Similarly, Li et al. [39] reported a Co_9_S_8_@MoS_2_ heterojunction material with pronounced energy-level separation. The impurity energy levels within the bandgap of Co_9_S_8_ were optimized, which enhanced its metallic characteristics and electrical conductivity. Beyond electronic modulation, heterojunctions also contribute to suppressing the shuttle effect, strengthening interfacial interactions, and enabling synergistic functionalities. Zhou et al. [40] developed a TiO_2_-TiN heterojunction as a dense and ultrathin separator coating, which effectively restricts LiPS shuttling even under high sulfur loading, enabling seamless LiPS capture–diffusion–conversion across interfaces. Ren et al. [41] engineered 2D/2D Ti_3_C_2_T_x_/Ni-Co MOF heterojunction nanosheets coated on separators, achieving dual functions of shuttle suppression and synergistic adsorption–electrocatalysis. Yao et al. [42] integrated SnS (with strong adsorption and high conductivity) and ZnS (with superior catalytic activity) into a ZnS-SnS@NC heterojunction encapsulated by an N-doped carbon shell. This architecture exhibited enhanced lithiophilic and sulfiphilic properties, significantly suppressing the shuttle effect while improving sulfur utilization and cycle stability. Lei et al. [43] designed a MoS_2_-MoO_3_ heterojunction nanosheet that shortens Li⁺/electron transport pathways. Its strong donor–acceptor interactions enhance interfacial electrochemical reactions, while the built-in electric field accelerates charge transfer and LiPS conversion. Moreover, the synergistic interplay between MoO_3_ and MoS_2_ provides abundant adsorption/catalytic sites, further mitigating the shuttle effect and boosting sulfur utilization. Despite these advances, individual components in heterojunctions often struggle to integrate multiple advantages, such as abundant catalytic active sites, robust chemical adsorption, high conductivity, and rapid Li⁺ diffusion. Theoretical insights suggest that rational heterojunction design could holistically unify these merits, paving the way for next-generation electrocatalytic systems with unprecedented performance [44].

In this study, we investigated the catalytic and adsorption mechanisms of metal nitride heterostructure toward lithium polysulfides (LiPSs). The Ni_3_N/Nb_4_N_5_ heterostructure exhibits a uniform dispersion and porous architecture, which facilitates ion transport and provides abundant active sites for LiPS conversion. The visualization of adsorption tests and X-ray photoelectron spectroscopy (XPS) analysis confirmed the strong chemisorption capability of Ni_3_N/Nb_4_N_5_ toward LiPSs. Furthermore, the constructed heterogeneous interface facilitates accelerated charge transfer kinetics, while the synergistic catalytic effect between bimetallic nitrides promotes the homogeneous nucleation and growth of Li_2_S. Electrochemical evaluations demonstrate that the Ni_3_N/Nb_4_N_5_-PP-based cell achieved enhanced cycling stability and rate capability, delivering discharge capacities of 1403.6, 1032.4, 909.5, and 793.9 mAh g^−1^ at 0.1 C, 0.25 C, 0.5 C, and 1.0 C, respectively. Notably, the Li-S cell retained a discharge capacity of 796.2 mAh g^−1^ after 150 cycles at 0.2 C.

## 2. Experimental Procedures

### 2.1. Materials

Niobium oxalate hydrate: Alfa Aesar Chemical Company (Shanghai, China); N,N-dimethylformamide: Sinopharm Chemical Reagent Company (Shanghai, China); nickel acetate tetrahydrate: Sinopharm Chemical Reagent Company (Shanghai, China); peroxyacetyl nitrate: Aladdin ReagentCompany (Shanghai China); electrolyte: DuoDuo Chemical Reagent (Suzhou, China); polyvinylidene fluoride: Aldrich Chemical Company (Shanghai, China); N-methylpyrrolidone: Sigma Chemical Reagent Company (Shanghai, China).

### 2.2. Fabrication of Ni₃N/Nb₄N₅ Heterostructure

The fabrication of the Ni_3_N/Nb_4_N_5_ heterostructure was achieved through a sequential process that entailed the combination of electrospinning and controlled thermal treatments. The synthesis protocol that was utilized is outlined as follows:

Precursor solution preparation: The formation of a homogeneous solution was achieved through the dissolution of 2 mmol of niobium oxalate hydrate (C_10_H_5_NbO_20_) in 4 mL of DMF, under constant stirring at 60 °C. Subsequently, 1 mmol of nickel acetate tetrahydrate (Ni(CH_3_COO)_2_·4H_2_O) was added, and the mixture was stirred for a further 8 h.

Polymer matrix preparation: Concurrently, 1 g of polyacrylonitrile (PAN) was dissolved in 6 mL of DMF, with the solution being subjected to mechanical stirring at ambient temperature for a duration of 8 h.

Electrospinning process: The two solutions were thoroughly blended prior to processing through the electrospinning apparatus.

Thermal conversion: The electrospun fibers were then subjected to oxidative calcination at 800 °C (with a 5 °C/min ramp rate) for a duration of three hours in air, with the objective of producing the NiNb_2_O_6_ intermediate.

Nitridation: The final transformation was achieved by subjecting the NiNb_2_O_6_ precursor to ammoniating at 800 °C for 3 h (with a heating rate of 5 °C/min) under NH_3_/Ar flow, thereby yielding the desired Ni_3_N/Nb_4_N_5_ nanocomposite.

### 2.3. Preparation of Modified Separators

The electrode slurry was prepared by means of homogenously blending active materials (Ni_3_N/Nb_4_N_5_ heterostructure or alternatively NiNb_2_O_6_), a conductive additive (Super P carbon black), and a polymeric binder (PVDF) in an 8:1:1 mass ratio through mechanical grinding. The subsequent addition of N-methyl-2-pyrrolidone (NMP) as a dispersion medium enabled uninterrupted magnetic stirring for a period of three hours, thereby achieving a well-dispersed slurry. The slurry was uniformly coated onto a polypropylene (PP, Celgard 2400) separator using a 200 μm coating blade with a coating machine. Subsequently, the modified separator was dried in a vacuum oven at 50 °C for 12 h. Finally, the separator was punched into 19 mm diameter disks, with an active material (Ni_3_N/Nb_4_N_5_ or NiNb_2_O_6_) loading of 1.3–1.9 mg cm^−2^. The Ni_3_N/Nb_4_N_5_ and NiNb_2_O_6_ modified separators were abbreviated as Ni_3_N/Nb_4_N_5_-PP and NiNb_2_O_6_-PP.

### 2.4. Visualized Adsorption of Polysulfides

This section will deal with the Li_2_S_6_ solution synthesis. The solvent system was initially prepared by combining 1,3-dioxolane (DOL) and dimethoxyethane (DME) at a volumetric ratio of 1:1. Subsequently, stoichiometric amounts of Li_2_S and elemental sulfur (in a 1:5 molar ratio) were dissolved in the DOL/DME mixture. The reaction system was maintained at a temperature of 60 °C for a period of 24 h, with the protection of argon, thereby yielding a solution of Li_2_S_6_ at a concentration of 5 mmol L^−1^. This solution was then diluted to a concentration of 2 mmol L^−1^. For the adsorption tests, 5 mL aliquots of the diluted solution were separately mixed with 10 mg portions of Ni_3_N/Nb_4_N_5_ and NiNb_2_O_6_ powders in an inert environment. The mixtures were maintained for a duration of 0~24 h to monitor their adsorption behavior, and images were captured at designated time intervals for further analysis.

### 2.5. Fabrication and Characterization of Symmetric Cells

The active material (Ni_3_N/Nb_4_N_5_ or NiNb_2_O_6_), conductive carbon black, and PVDF binder were then homogenously mixed in ethanol with a weight ratio of 8:1:1 under ultrasonication for 30 min to form a uniform electrode slurry. The homogenized slurry was uniformly deposited onto 12 mm diameter carbon fiber paper (Toray TGP-H-060,Toray Investment Co., Ltd., Shanghai, China)) using a micropipette, followed by drying at 50 °C for 12 h in a vacuum oven.

A CR2025 coin cell was assembled using a polypropylene (Celgard-2400) separator and 50 μL of Li_2_S_6_ electrolyte. The electrolyte was formulated by dissolving 1.0 M Li_2_S_6_ and 1.0 M LiTFSI in a 1:1 (*v*/*v*) binary solvent system comprising DOL and DME. The present study investigates the electrochemical behavior of LiPSs. To this end, cyclic voltammetry (CV) measurements were performed on the symmetric cell at scan rates of 5 and 50 mV s^−1^. The potential window was set between −0.8 and 0.8 V.

### 2.6. Experimental Procedure for Li₂S Nucleation Studies

The nucleation behavior was investigated using CR2025-type coin cells. Carbon paper substrates were coated with Ni_3_N/Nb_4_N_5_ or NiNb_2_O_6_ active materials, achieving mass loadings between 1.0 and 1.3 mg cm^−2^. Lithium foil acted as the counter electrode. In the context of the electrolyte system, the cathodic compartment was constituted of 0.25 M Li_2_S_6_ in conjunction with 1.0 M LiTFSI within a tetraethylene glycol dimethyl ether solvent, while the anodic compartment utilized a 1.0 M LiTFSI solution within an equivalent solvent. Precise 20 μL aliquots of each electrolyte solution were dispensed into their respective cell chambers. Electrochemical conditioning was initiated with a constant-current discharge at 2.06 V (0.112 mA). This was followed by a potentiostatic hold at 2.05 V, which was maintained until the current stabilized below 10^−5^ A. This process was found to promote Li_2_S crystallization [45].

### 2.7. Material Characterization

The crystalline structure was characterized by X-ray diffraction (XRD) analysis performed on a Bruker D8 Advance diffractometer equipped with a Cu Kα radiation source (wavelength = 1.54056 Å). The morphology and microstructure of the samples were characterized by scanning electron microscopy (SEM, JSM-7800F) and transmission electron microscopy (TEM, FEI Talos F200X G2, FEI Czech Ltd., Hillsboro, OR, USA), both equipped with energy-dispersive spectroscopy (EDS). The chemical composition of the sample was investigated through the implementation of X-ray photoelectron spectroscopy (XPS) analysis.

### 2.8. Material Preparation and Electrochemical Characterization

The sulfur/acetylene black (S/AB) composite was fabricated through mechanical ball-milling of sublimed sulfur and AB (3:2 mass ratio) for 2 h, followed by thermal treatment in a sealed reactor at 155 °C for 12 h. This facilitated sulfur melting and subsequent homogeneous distribution. Subsequent to cooling to ambient temperature, cathode slurries were prepared by blending the composite with PVDF binder (9:1 mass ratio) in NMP solvent. The homogeneous mixture was applied to carbon-modified Al current collectors by doctor-blading, followed by vacuum drying at 50 °C for 12 h. The electrodes were punched into 12 mm diameter disks, with sulfur loadings ranging from 1.0 to 1.5 mg cm^−2^. The assembly of coin cells was conducted within an Ar atmosphere glovebox, utilizing lithium metal anodes, prepared S/AB cathodes, and Ni_3_N/Nb_4_N_5_-PP (or an alternative) separators. The electrolyte formulation consisted of 1.0 M LiTFSI and 2 wt% LiNO_3_ in DME (1:1 *v*/*v*) solvent, with strict control of electrolyte volume at 30 μL per mg sulfur. Galvanostatic charge–discharge tests were performed on a LAND battery testing system within a voltage window of 1.7–2.8 V. Cyclic voltammetry (CV) was conducted at a scan rate of 0.1 mV s^−1^, and electrochemical impedance spectroscopy (EIS) was performed over a frequency range of 0.01 Hz to 100 kHz.

## 3. Results and Discussion

**Figure 1:**
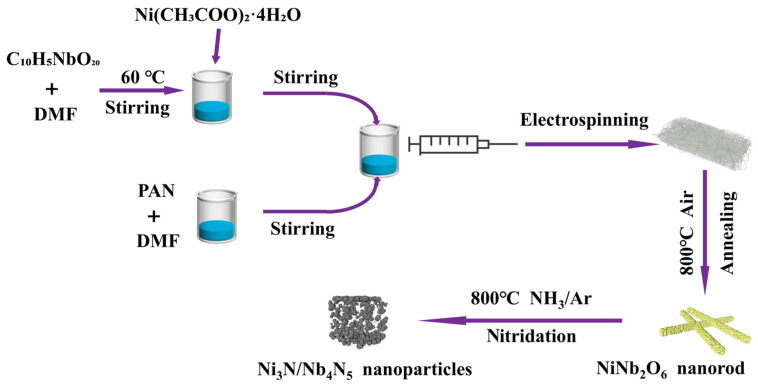
Schematic diagram of the preparation of Ni_3_N/Nb_4_N_5_ heterostructure.

Figure 1 illustrates the preparation process of the NiNb_2_O_6_ nanorods and the Ni_3_N/Nb_4_N_5_ heterostructure. Initially, NiNb_2_O_6_ nanorods, composed of uniformly sized nanoparticles, were synthesized via the electrospinning method, demonstrating high homogeneity. Subsequently, the NiNb_2_O_6_ nanorods underwent nitridation treatment in an ammonia atmosphere to form the Ni_3_N/Nb_4_N_5_ heterostructure. After nitridation, the nanorods exhibited a well-dispersed morphology, leading to the formation of homogeneous Ni_3_N/Nb_4_N_5_ nanoparticles.

**Figure 2:**
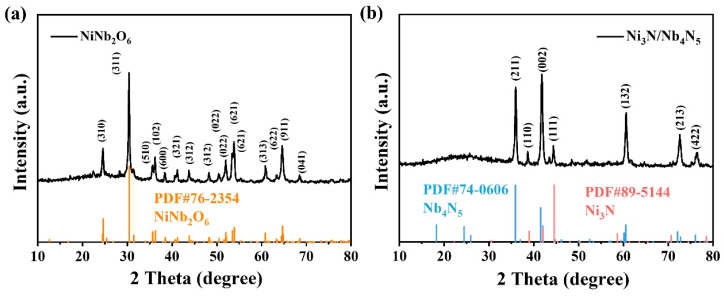
(**a**) XRD patterns of NiNb_2_O_6_ nanorods and (**b**) Ni_3_N/Nb_4_N_5_ heterostructure.

The crystal structure of the as-prepared NiNb_2_O_6_ and Ni_3_N/Nb_4_N_5_ heterostructure were analyzed and verified by XRD. As shown in Figure 2a, several typical diffraction peaks in the XRD pattern of NiNb_2_O_6_ are in good agreement with the reference spectrum of NiNb_2_O_6_ nanorods (JCPDS No. 76-2354), confirming the successful synthesis of crystalline NiNb_2_O_6_. In addition, Figure 2b shows the XRD pattern of the Ni_3_N/Nb_4_N_5_ heterostructure, where the major diffraction peaks correspond to Nb_4_N_5_ (JCPDS No. 74-0606) and Ni_3_N (JCPDS No. 89-5144). The presence of non-impurity peaks indicates that the pure Ni_3_N/Nb_4_N_5_ heterostructure was successfully synthesized through the nitridation of NiNb_2_O_6_.

**Figure 3:**
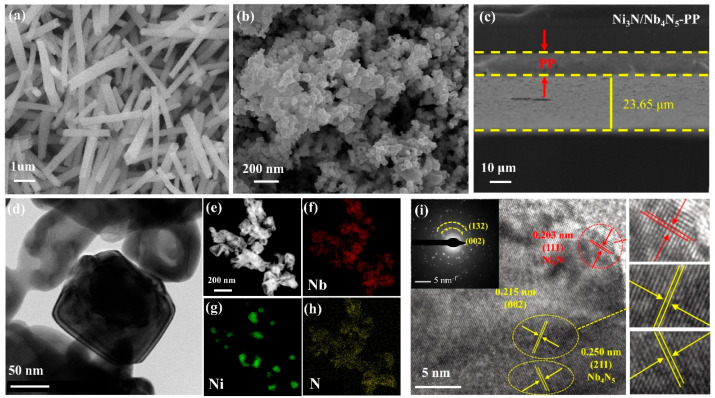
SEM image of (**a**) NiNb_2_O_6_ and (**b**) Ni_3_N/Nb_4_N_5_ heterostructure; (**c**) cross-sectional SEM of Ni_3_N/Nb_4_N_5_-PP; (**d**) low-magnification TEM image of Ni_3_N/Nb_4_N; (**e**–**h**) STEM image and EDS elemental mappings; (**i**) high-resolution TEM image of Ni_3_N/Nb_4_N.

The microstructure of NiNb_2_O_6_ and Ni_3_N/Nb_4_N_5_ heterostructure, as well as the cross-section of the modified separator, were observed using scanning electron microscopy (SEM). As shown in Figure 3a, the NiNb_2_O_6_ exhibits a regular nanorod structure. In contrast, the nitrided Ni_3_N/Nb_4_N_5_ heterostructure primarily displays a nanoparticle structure (Figure 3b), with uniform dispersion and good homogeneity. Moreover, the nanoparticle stacking structure features a large surface area, allowing the space between nanoparticles to be effectively infiltrated and stored with electrolyte, thereby enhancing the electron exchange rate [46]. A slurry was prepared to coat the commercial PP separator, and the thickness of the coated layer was approximately 23.65 μm, as observed in the cross-sectional view (Figure 3c). Furthermore, the TEM and EDS mapping in Figure 3d–h confirm the presence of Nb, Ni, and N elements in the Ni_3_N/Nb_4_N_5_ heterostructure, indicating the successful incorporation of these elements. As demonstrated in Figure 3i, high-resolution TEM analysis clearly identified two characteristic interplanar distances measuring 0.250 nm and 0.203 nm, respectively. These were indexed to the (211) crystallographic plane of Nb_4_N_5_ and the (111) plane of Ni₃N, thus confirming the coexistence of both nitride phases. Additionally, a lattice spacing of 0.215 nm was observed, which corresponds to the (002) plane present in both Ni_3_N and Nb_4_N_5_. These findings are consistent with the reference data for Nb_4_N_5_ (JCPDS No. 74-0606) and Ni_3_N (JCPDS No. 89-5144) at 42° in the XRD spectra. Therefore, the Ni_3_N/Nb_4_N_5_ heterostructure was successfully synthesized through the nitridation of NiNb_2_O_6_.

**Figure 4:**
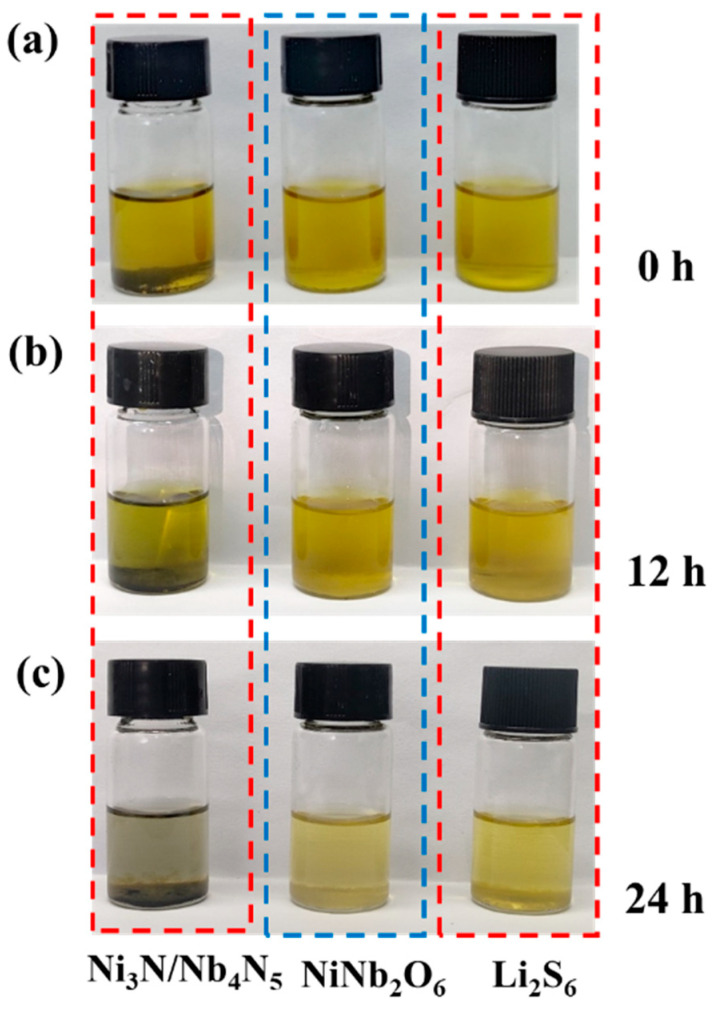
(**a**–**c**) Visual adsorption experiment of Li_2_S_6_ by pristine Ni_3_N/Nb_4_N_5_ and NiNb_2_O_6_.

In order to undertake a systematic investigation into the immobilization performance of LiPSs of the Ni_3_N/Nb_4_N_5_ composite, comparative visual adsorption experiments were performed under controlled conditions. The quantitative results were then documented visually, as illustrated in Figure 4a–c. After 12 h of standing, the yellow color of the Li_2_S_6_ solution containing Ni_3_N/Nb_4_N_5_ powder partially faded, whereas the solution with NiNb_2_O_6_ powder exhibited almost no color change. After 24 h, the yellow color of the Li_2_S_6_ solution with Ni_3_N/Nb_4_N_5_ powder completely disappeared, while the solution containing NiNb_2_O_6_ showed only slight fading [47,48]. These results demonstrate that Ni_3_N/Nb_4_N_5_ exhibits a significantly stronger adsorption capability for LiPSs compared to NiNb_2_O_6_.

**Figure 5:**
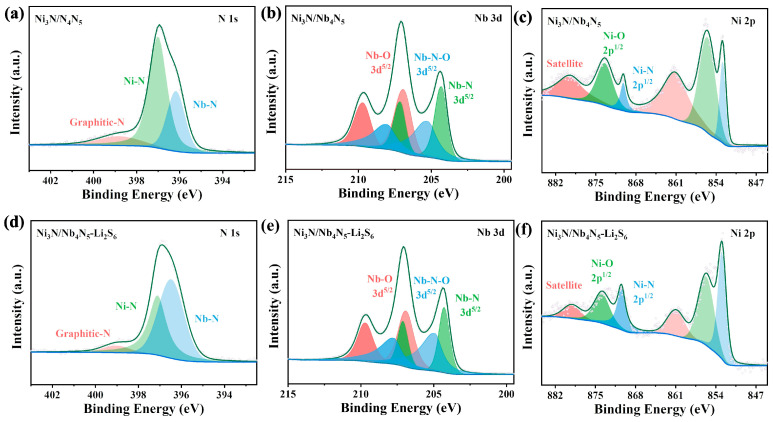
High-resolution XPS spectra of (**a**) N 1s, (**b**) Nb 3d, and (**c**) Ni 2p before Ni_3_N/Nb_4_N_5_ adsorption of Li_2_S_6_, and (**d**) N 1s, (**e**) Nb 3d, and (**f**) Ni 2p after Ni_3_N/Nb_4_N_5_ adsorption of Li_2_S_6_.

To further investigate the interaction mechanism between Ni_3_N/Nb_4_N_5_ and LiPSs, XPS analysis was conducted on the samples before and after Li_2_S_6_ adsorption. Figure 5 presents the high-resolution XPS spectra of N 1s, Nb 3d, and Ni 2p before and after Li_2_S_6_ adsorption by Ni_3_N/Nb_4_N_5_. In Figure 5a,d, the N 1s spectra exhibit three characteristic peaks corresponding to the Ni-N bond, Nb-N bond, and graphitic nitrogen. Following the adsorption of Li_2_S_6_, a shift in the peak positions of the three characteristic peaks was observed. The original peak positions of 397.03 eV/396.14 eV/398.84 eV shifted to higher binding energies of 0.11 eV, 0.31 eV, and 0.22 eV, respectively. In Figure 5b–e, the Nb 3d spectra display three pairs of characteristic peaks, assigned to Nb-O, Nb-N-O, and Nb-N bonds. The oxygen signal likely originates from surface oxidation due to exposure to air [37]. After the adsorption of Li_2_S_6_, the binding energies of Nb-O and Nb-N showed negligible changes; however, the Nb-N-O binding energy shifted to the right by 0.32 eV from the original 208.18 eV/205.38 eV. Previous studies have demonstrated that surface oxide layers on metal compounds can activate surface metal sites through strong chemical bonds for polysulfide bonding [49]. Similarly, the Ni 2p spectra (Figure 5c,f) show three pairs of characteristic peaks, including satellite peaks, Ni-O bonds, and Ni-N bonds. After Li_2_S_6_ adsorption, the binding energies of the Ni-O and Ni-N bonds increased by 0.45 eV and 0.29 eV, respectively.

**Figure 6:**
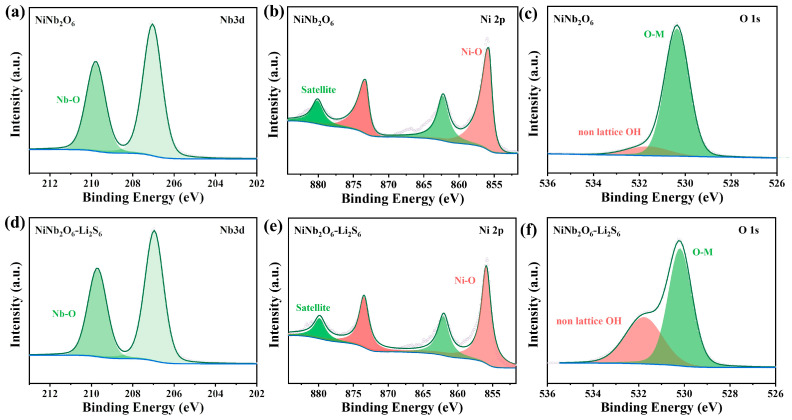
High-resolution XPS spectra of (**a**) Nb 3d, (**b**) Ni 2p, and (**c**) O 1s before NiNb_2_O_6_ adsorption of Li_2_S_6_, and high-resolution XPS spectra of (**d**) Nb 3d, (**e**) Ni 2p, and (**f**) O 1s after NiNb_2_O_6_ adsorption of Li_2_S_6_.

Compared to the Ni_3_N/Nb_4_N_5_ heterostructure, the XPS high-resolution spectra of Nb 3d, O 1s [50,51], and Ni 2p [52,53] before/after the NiNb_2_O_6_ adsorption of Li_2_S_6_ are shown in Figure 6. No significant change in binding energy was observed before and after adsorption, indicating that Ni_3_N/Nb_4_N_5_ exhibited stronger chemical adsorption capability for lithium LiPSs. This enhanced adsorption effectively suppressed the shuttle effect in Li-S batteries and improved the utilization of sulfur.

**Figure 7:**
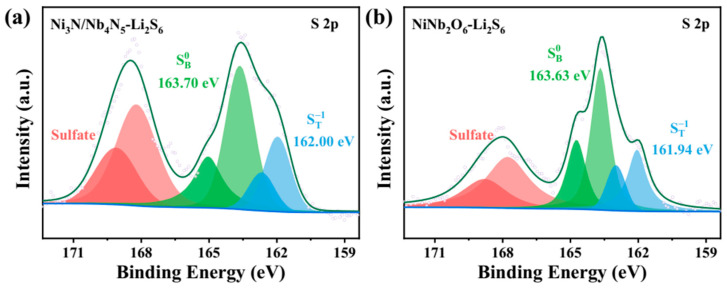
High-resolution XPS spectra of S 2p after adsorption of Li_2_S_6_ by (**a**) Ni_3_N/Nb_4_N_5_ heterojunction and (**b**) NiNb_2_O_6_.

In addition, the high-resolution S 2p spectra of both Ni_3_N/Nb_4_N_5_ and NiNb_2_O_6_ (Figure 7a,b) exhibit three pairs of characteristic peaks. The binding energy of terminal sulfur $S_{T}^{-1}$ in Ni_3_N/Nb_4_N_5_ (162.00 eV) was slightly higher than that in NiNb_2_O_6_ (161.94 eV). Similarly, the binding energy of bridging sulfur ($S_{B}^{0}$) in Ni_3_N/Nb_4_N_5_ (163.70 eV) was higher than that in NiNb_2_O_6_ (163.63 eV). Additionally, the peak observed at 169.02 eV for Ni_3_N/Nb_4_N_5_ can be assigned to thiosulfate species [54]. Compared with NiNb_2_O_6_, strong thiosulfate signals were detected in Ni_3_N/Nb_4_N_5_, which could promote the conversion of higher-order LiPSs into short-chain lithium sulfides. This further demonstrates that Ni_3_N/Nb_4_N_5_ plays a critical role in enhancing the kinetics of sulfide conversion, whereas only weak thiosulfate signals were observed in NiNb_2_O_6_ [55]. As demonstrated by visual adsorption tests and XPS characterization, the heterogeneous interface of Ni_3_N/Nb_4_N_5_ facilitates charge transfer and exhibits enhanced thiophilicity, which promotes the adsorption of lithium LiPSs and effectively suppresses the shuttle effect in Li-S batteries.

**Figure 8:**
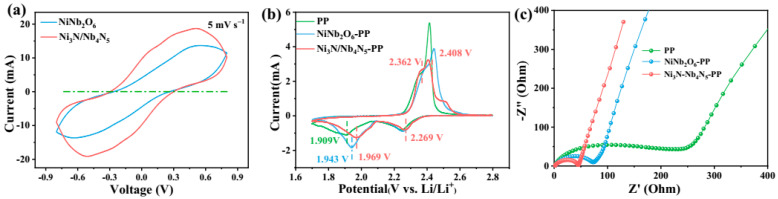
CV curves of (**a**) symmetric cells with Ni_3_N/Nb_4_N_5_ and NiNb_2_O_6_; (**b**) the initial CV curve and (**c**) EIS curve of Li-S cells with Ni_3_N/Nb_4_N_5_-PP, NiNb_2_O_6_-PP, and PP separators.

To investigate the effect of the Ni_3_N/Nb_4_N_5_-modified separator on the electrochemical performance of Li-S batteries, the samples were coated onto a commercial separator for modification and the Li-S cells were assembled for electrochemical testing. Figure 8a presents the cyclic voltammetry (CV) curves of symmetric cells with different electrodes at a scan rate of 5 mV s^−1^. It can be observed that the Ni_3_N/Nb_4_N_5_ electrode exhibits higher redox current peaks and smaller redox peak separation compared to the NiNb_2_O_6_ electrode, whereas the CV curve of the NiNb_2_O_6_ cell shows greater potential hysteresis. The initial cycle of CV tests conducted at a scan rate of 0.1 mV s^−1^ for the Li-S cells assembled with Ni_3_N/Nb_4_N_5_-PP, NiNb_2_O_6_-PP, and PP separators are shown in Figure 8b. The two reduction peaks observed at approximately 2.27 V and 1.97 V correspond to the conversion of S_8_ to long-chain LiPSs and to the final products Li_2_S_2_/Li_2_S, respectively. Additionally, the anodic peaks around 2.36 V and 2.48 V correspond to the conversion of Li_2_S_2_/Li_2_S to long-chain LiPSs and S_8_. The Ni_3_N/Nb_4_N_5_-PP separator exhibits three distinct electrochemical advantages in comparison to both NiNb_2_O_6_-PP and PP separators. The following observations were made: firstly, there was a shift in the reduction peaks toward higher potentials; secondly, there was a decrease in the overpotential gap; and thirdly, there was a shift in the oxidation peaks to lower potentials. This combination of effects led to significantly lower charge–discharge overpotentials. This electrochemical behavior indicates that the Ni_3_N/Nb_4_N_5_ heterostructure effectively promotes LiPS redox kinetics [56]. In order to conduct a more in-depth investigation into the interfacial characteristics, we undertook electrochemical impedance spectroscopy (EIS) measurements. The objective of this was to ascertain the charge transfer resistance (Rct) between the electrode and the electrolyte. As illustrated in Figure 8c, the resulting Nyquist plots for all cell configurations are presented. The results revealed that the Rct of the Ni_3_N/Nb_4_N_5_-PP cell (45.09 Ω) was significantly lower than that of the NiNb_2_O_6_-PP (72.12 Ω) and PP (242.1 Ω) cells, suggesting that Ni_3_N/Nb_4_N_5_ accelerates electron transfer and reaction kinetics at the electrode–electrolyte interface [57].

**Figure 9:**
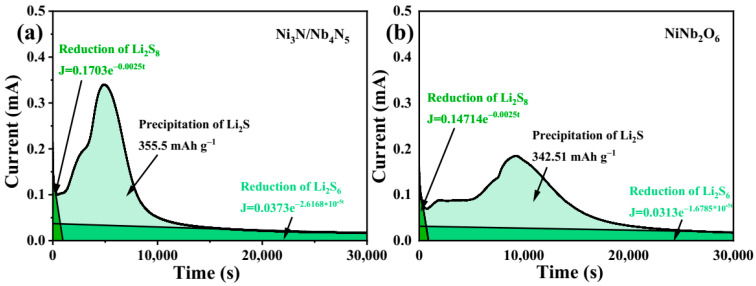
Potentiostatic discharge curves of (**a**) Ni_3_N/Nb_4_N_5_ and (**b**) NiNb_2_O_6_ electrode.

To investigate the capability of Ni_3_N/Nb_4_N_5_ nanoparticles in catalyzing the conversion of LiPSs Li_2_S, nucleation experiments were conducted. As illustrated in Figure 9a,b, the initial current drop is attributed to the reduction of higher-order LiPSs in the solution to Li_2_S_4_ [58]. The Ni_3_N/Nb_4_N_5_ sample exhibited a significantly shorter nucleation time (4800 s) compared to NiNb_2_O_6_ (9300 s), indicating faster kinetics of Li_2_S nucleation. Additionally, Ni_3_N/Nb_4_N_5_ demonstrated a stronger Li_2_S nucleation capability than NiNb_2_O_6_. This comparison suggests that the catalytic effect of Ni_3_N/Nb_4_N_5_ can lower the energy barrier for Li_2_S growth, thereby enhancing the conversion of soluble LiPSs to insoluble solid Li_2_S [59].

**Figure 10:**
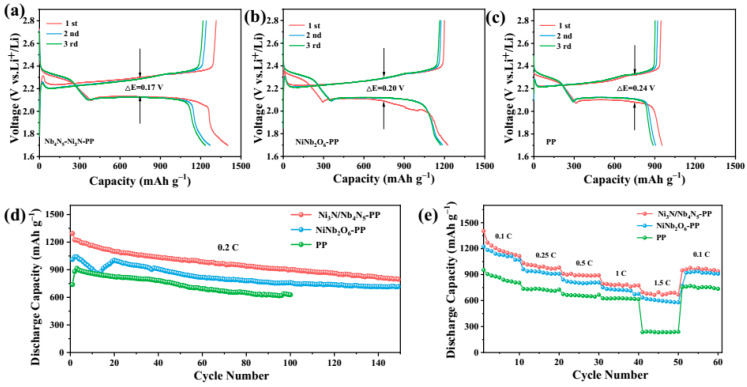
Charge–discharge curves of the first three cycles of Li-S cells with (**a**) Ni_3_N/Nb_4_N_5_-PP, (**b**) NiNb_2_O_6_-PP, and (**c**) PP at 0.1 C; (**d**) cyclic performance of Li-S cells with Ni_3_N/Nb_4_N_5_-PP, NiNb_2_O_6_-PP, and PP at 0.2 C; (**e**) rate performance.

Furthermore, the cycling stability of Li-S cells assembled with Ni_3_N/Nb_4_N_5_-PP, NiNb_2_O_6_-PP, and PP separators was evaluated. The materials under consideration were Ni_3_N/Nb_4_N_5_-PP, NiNb_2_O_6_-PP, and standard PP. Initial cycling tests conducted at 0.1 C rate revealed substantial differences in discharge capacities, with the Ni_3_N/Nb_4_N_5_-PP -PP cell achieving 1403.6 mAh g^−1^, outperforming both the NiNb_2_O_6_-PP (1224 mAh g^−1^) and unmodified PP (948.6 mAh g^−1^) counterparts. The corresponding charge–discharge profiles for the initial three cycles are illustrated in Figure 10a–c. It is important to note the observed potential difference at 800 mAh g^−1^ capacity, where the Ni_3_N/Nb_4_N_5_-PP system exhibited a minimal 0.17 V polarization, which is substantially lower than the 0.2 V and 0.24 V values recorded for the NiNb_2_O_6_-PP and PP systems, respectively. The reduced polarization suggests that the electrocatalytic activity of Ni_3_N/Nb_4_N_5_ decreases the kinetic resistance of Li-S batteries, with Li-S cells with Ni_3_N/Nb_4_N_5_-PP demonstrating low polarization and superior reversibility [14]. From the long-term cycling performance depicted in Figure 10d, it is evident that the Li-S cell with Ni_3_N/Nb_4_N_5_-PP exhibited an initial discharge capacity of 1294.4 mAh g^−1^ at 0.2 C, and after 150 cycles, the discharge capacity was retained at 796.2 mAh g^−1^, with a capacity decay rate of 0.25% per cycle. Electrochemical performance comparisons revealed significant differences among the tested separators. The NiNb_2_O_6_-PP configuration demonstrated an initial discharge capacity of 1011.9 mAh g^−1^, which is considerably lower than that of the Ni_3_N/Nb_4_N_5_-PP system. This observation serves to confirm the enhanced cycling stability of the latter. This performance discrepancy may originate from the inferior conductivity of NiNb_2_O_6_, as reported in previous studies [60,61]. It has been demonstrated that the Ni_3_N/Nb_4_N_5_-PP cells exhibit superior cycling stability. This can be attributed to the formation of heterostructured interfaces, which enhance electron transport kinetics and reduce polarization loss during cycling. Furthermore, the high electrical conductivity of Ni_3_N promotes the formation of a more homogeneous SEI layer, which inhibits the continuous decomposition of the electrolyte [62]. Testing the rate capability of the material across the range of 0.1–1.5 C (Figure 10e) demonstrated the exceptional performance of the Ni_3_N/Nb_4_N_5_-PP, with capacities of 1403.6 mAh g^−1^ (0.1 C), 1032.4 mAh g^−1^ (0.25 C), 909.5 mAh g^−1^ (0.5 C), and 793.9 mAh g^−1^ (1.0 C) being maintained. It is noteworthy that even at the maximum 1.5 C rate, this system demonstrated a capacity of 695.4 mAh g^−1^, which surpassed the capacities of both NiNb_2_O_6_-PP (634.5 mAh g^−1^) and PP (235.7 mAh g^−1^) systems. Upon returning to 0.1 C, the Ni_3_N/Nb_4_N_5_-PP cell exhibited a recovery of 949.4 mAh g^−1^, thereby further substantiating its superior rate adaptability and capacity retention in comparison to alternative configurations.

## 4. Conclusions

In this study, the Ni_3_N/Nb_4_N_5_ heterostructure was synthesized using electrospinning followed by nitridation treatment and applied as a modified separator for Li-S batteries. The Ni_3_N/Nb_4_N_5_ heterostructure was uniformly dispersed and possessed abundant porosity, which facilitated ion transport. The visual adsorption tests and XPS results demonstrated that the Ni_3_N/Nb_4_N_5_ had a strong adsorption capability for LiPSs, and the heterointerface promoted accelerated charge transfer. The Li_2_S nucleation tests confirmed that the Ni_3_N/Nb_4_N_5_ enhanced the nucleation and growth of Li_2_S. The electrochemical measurements revealed that the Li-S cell with Ni_3_N/Nb_4_N_5_-PP could deliver an initial discharge capacity of 1294.4 mAh g^−1^ at 0.2 C, with a capacity decay rate of 0.25% per cycle after 150 cycles, demonstrating superior cycling stability and rate performance compared to the Li-S cells with NiNb_2_O_6_-PP and pristine separator. This study introduces a novel method for fabricating the Ni_3_N/Nb_4_N_5_ heterostructure, which markedly enhances the electrochemical performance of Li-S batteries.

## Figures and Tables

**Table 1:** Some recent heterojunction applications for Li-S battery interlayers.

		Typology	Current Density	Number of Cycles	Capacity Decay Rate	Capacity/Retention Rate
1	Co_9_S_8_@MoS_2_/CNF	interlayer	1.0 C	400	0.09%	[39]
2	TiO_2_–TiN	interlayer	0.3 C	300	/	927 mAh g^−1^ [40]
3	Ti_3_C_2_T_x_/Ni-Co MOF	modified separators	0.2 C	200	0.06%	1100 mAh g^−1^/87.3% [41]
4	ZnS-SnS@NC	modified separators	4.0 C	2000	0.01%	632 mAh g^−1^/74.9% [42]
5	MoS_2_-MoO_3_	modified separators	1.0 C	600	0.01%	92.00% [43]
6	This work	modified separators	0.2 C	200	0.25%	796.2 mAh g^−1^

## Data Availability

The data that support the findings of this study are available from the corresponding author upon reasonable request.
